# Postbiotics against Pathogens Commonly Involved in Pediatric Infectious Diseases

**DOI:** 10.3390/microorganisms8101510

**Published:** 2020-09-30

**Authors:** Anastasia Mantziari, Seppo Salminen, Hania Szajewska, Jeadran Nevardo Malagón-Rojas

**Affiliations:** 1 Functional Foods Forum, Faculty of Medicine, University of Turku, 20520 Turku, Finland; sepsal@utu.fi; 2 Department of Paediatrics at the Medical University of Warsaw, 02091 Warsaw, Poland; hania@ipgate.pl; 3 Facultad de Medicina, Universidad El Bosque, 110121 Bogotá, Colombia; jnmalagon@unbosque.edu.co; 4 Instituto Nacional de Salud de Colombia, 111321 Bogotá, Colombia

**Keywords:** postbiotics, probiotics, common infectious diseases, children, public health

## Abstract

The Sustainable Development goals for 2020 included reducing all causes associated with infant and perinatal mortality in their priorities. The use of compounds with bioactive properties has been proposed as a therapeutic strategy due to their stimulating effect on the host’s immune system. Additionally, biotherapeutic products such as postbiotics, tentatively defined as compounds produced during a fermentation process that support health and well-being, promote intestinal barrier integrity without posing considerable risks to children’s health. Although this is a concept in development, there are increasing studies in the field of nutrition, chemistry, and health that aim to understand how postbiotics can help prevent different types of infections in priority populations such as minors under the age of five. The present review aims to describe the main mechanisms of action of postbiotics. In addition, it presents the available current evidence regarding the effects of postbiotics against pathogens commonly involved in pediatric infections. Postbiotics may constitute a safe alternative capable of modulating the cellular response and stimulating the host’s humoral response.

## 1. Introduction

Saving children’s lives and improving child health are two critical global priorities and represent essential targets of Goal 3 (Good Health and Well-Being) of the United Nations Sustainable Development Goals. Since 1990, there has been a significant reduction in the mortality rates of children aged one month to five years old [1]. However, this is not the case for newborn mortality rates that are falling behind, accounting for the more significant proportion of all deaths among children younger than five years old. To understand this pattern, one must look closer at the causes of newborn deaths. The vast majority of infant deaths occur within the first month of life and are caused by being born too soon, complications during delivery, and infectious diseases [2]. Among infectious diseases, diarrheal disease is one of the leading causes of death in children under five years old [3]. Common causes of bacterial diarrhea include diarrhoeagenic *Escherichia coli* (DEC; *E. coli*), *Shigella* species, *Salmonella* species, *Campylobacter*, and *Yersinia enterocolitica*. Besides *E. coli*, rotavirus (RV) is the other most common etiological agent of moderate-to-severe diarrhea in low-income countries. Fortunately, most of these causes are preventable and treatable but require global attention, increased access to quality healthcare, and cost-effective nutritional interventions.

Various factors contribute to infectious disease risk, including low birth weight, genetics, and diet, among others. The establishment of a healthy gut microbiome plays a pivotal role in the development of the immune system and could potentially lower the risk of infectious diseases in infants and children. One way to shape and modulate the gut microbiome is through diet. Although breast milk is the optimal source of nutrition for developing a diverse and healthy microbiota composition, there are cases that it may not always be available or adequate for the infant. For this reason, infant formulas are manufactured and are continuously being improved as they aim to resemble the nutritional composition of breast milk. Over the past decades, different preparations and interventions, such as supplementation with probiotics, have been explored, aiming to improve the composition of infant formulas. Probiotics are defined as ‘’live microorganisms that, when administered in adequate amounts, confer a health benefit on the host’’ [4]. The rationale for using probiotics to fight infections is that probiotics demonstrate a good ability to bind to intestinal mucus and compete with specific pathogens for the same adhesion sights. They therefore block pathogen adherence and consequently make pathogen propagation challenging [5]. The adhesion of probiotics to intestinal mucus or epithelial cells is mediated by surface proteins of probiotic bacteria and can inhibit pathogen adhesion through competitive exclusion. Some of these bacterial surface compounds include proteins, glycoproteins, lipoproteins, lipoteichoic acids, lipopolysaccharides, adhensins, and flagellins [6]. These properties support supplementing infant formulas with probiotics. However, at present, there is insufficient data to recommend the routine use of probiotic-supplemented formulas [7,8,9].

Besides providing possible protection against infections, probiotics may be a good new alternative antimicrobial solution when an infant is already infected by a pathogen. One intervention aimed to treat bacterial diarrhea is antibiotic therapy, although it is generally not recommended (with some exceptions). Moreover, many pathogenic strains have developed resistance to antibiotics [10]. On the other hand, there is the increasing concern that some probiotics harvest antibiotic-resistant genes in their genome that can be transferred to other potentially pathogenic bacteria [11,12]. In addition, many physicians are still skeptical with regards to the use of probiotics for pediatric care due to rare case reports of probiotic adverse effects [13,14]. Growing evidence suggests that many probiotic strains do not have to be alive to exert potential beneficial effects on host health [15]. For instance, it has been shown that, depending on the inactivation method, inactivated probiotics can adhere better to intestinal mucus compared to viable bacteria [16]. On the basis of these facts, new research is exploring the use of fermented infant formulas containing inactivated probiotics and/or their metabolic products and their role in protecting against infections [17,18,19].

Recently, new terms such as postbiotics, paraprobiotics, metabiotics, proteobiotics, pharmabiotics, and ghost probiotics have emerged [20,21,22,23,24,25]. Interestingly, evidence from the literature suggests that by using the term postbiotics, one refers to either inactivated probiotic strains or their metabolic products or both. Currently, a definition is under development; however, tentatively, postbiotics have been defined as bioactive ingredients from food-grade microorganisms resulting from a fermentation process that supports health and/or well-being [26]. The term postbiotic includes inactivated microbes and/or cell structures or metabolites that are released after bacterial lysis or that are secreted during fermentation. In most studies, postbiotics derive from the fermented culture medium that is being filtered or heated after the growth of the microorganism, resulting in a liquid called cell-free supernatant (CFS; known also as spent culture supernatant (SCS) or cell-free spent medium (CFSM)) [27]. This means that a combination of bioactive molecules is present rather than one specific purified compound [27]. Other ways to obtain postbiotics include the inactivation of probiotics using heat, filtration, sonication, centrifugation, and ultraviolet radiation (UV), among others [28]. In this case, bacterial lysis can occur, releasing a large array of compounds such as DNA, enzymes, lipoteichoic acids, and other intracellular metabolites that can serve as possible postbiotics [28].

A 2020 systematic review evaluated the clinical effects of postbiotics for preventing and treating common infectious diseases in children [29]. Given that there is limited research on postbiotics, coupled with their potential beneficial effects, this review investigates and reports the possible preventive mechanisms and uses of postbiotics against pediatric infectious diseases on the basis of current evidence. 

## 2. Potential Mechanisms of Action

**Figure 1:**
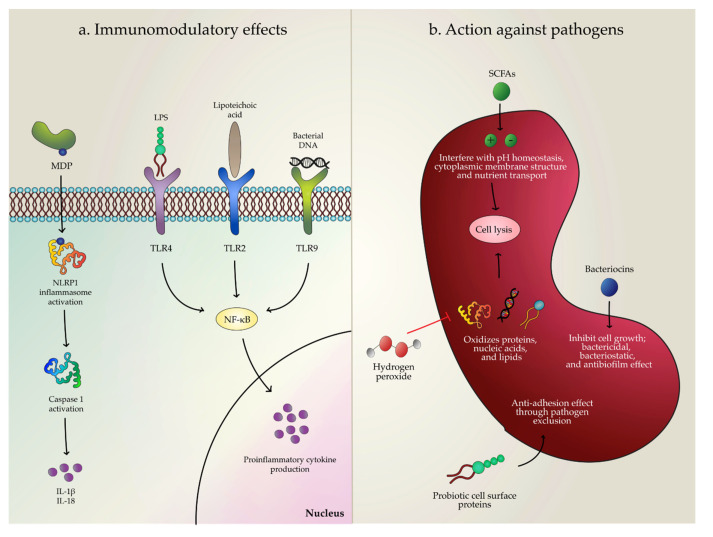
Potential mechanisms of action of postbiotics. (**a**) Modulation of the host’s immune response. (**b**) Acting on the pathogen. MDP: muramyl dipeptide; NLRP1: NACHT [NAIP (neuronal apoptosis inhibitory protein), CIITA (MHC class II transcription activator), HET-E (incompatibility locus protein from *Podospora anserina*) and TP1 (telomerase-associated protein)] domain-, leucine-rich repeat-, and PYRIN containing protein 1; IL-1β and IL-18: interleukin 1 beta and 18, respectively; LPS: lipopolysaccharide; TLR 4, TLR 2, and TLR 9: toll-like receptor 4, 2, and 9, respectively; NF-κB: nuclear factor kappa B; SCFAs: short-chain fatty acids.

The action mechanism of postbiotics and their role in improving host health are not yet clearly defined. However, two mechanisms might explain how postbiotics can stimulate and modulate the host’s immunological response, involving both elicit immune and acquired host response. The initial response is related to the innate immune system, and it consists of a series of pattern recognition receptors able to associate with microorganisms. Two of these pattern recognition receptors involved in the host response to postbiotics are the nucleotide-binding and oligomerization domain (NOD)-like receptors (NLRs) and the toll-like receptors (TLRs) (Figure 1).

The NOD domain of the NLRs functions as an intracellular receptor and is associated with the innate immune response [30]. On the basis of their terminal domain, NLRs are divided into four functional categories: inflammasome assembly, signaling transduction, transcription activation, and autophagy [31]. The NLRs can recognize different ligands from microbial pathogens such as viral RNA; peptidoglycan; and flagellin, the main component of flagella. For instance, muramyl dipeptide (MDP), a bioactive peptidoglycan motif common to all bacteria, activates the NACHT [(which comes from present in NAIP (neuronal apoptosis inhibitory protein), CIITA (MHC class II transcription activator), HET-E (incompatibility locus protein from *Podospora anserina*) and TP1 (telomerase-associated protein)] domain-, leucine-rich repeat-, and PYRIN-containing protein 1 (NLRP1) [32]. Upon activation, NLRP1 forms a multi protein complex known as the NLRP1 inflammasome that is expressed in innate and adaptive immunity cells including epithelial cells, T lymphocytes, macrophages, and dendritic cells (DCs) [33]. This allows the activation of caspase-1, leading to the secretion of pro-inflammatory cytokines interleukin (IL)-1β and IL-18.

The host’s innate immune system can also be stimulated via activation of the TLRs, a family of receptors that can recognize associated pathogens [34]. Each type of TLR can bind to a specific bacterial structure: lipopolysaccharides (LPS) are recognized by TLR4; lipoproteins, lipoteichoic acid, and peptidoglycan are recognized by TLR2; and flagellin is recognized by TLR5 [35]. Moreover, TLR3, TLR7, and TLR8 can bind bacterial RNA, in contrast to TLR9, which has been identified as the DNA-recognizing receptor [36]. Altogether, the activation of TLRs modulates the cytokine profile (signaling molecules) in immune cells. For instance, TLR9 is responsible for recognizing bacterial CpG site (cytosines followed by guanine residues) DNA released from probiotics. Since the C+G content between bacterial species is different, their affinity to bind to and activate TLR9 may vary [37]. It is also worth mentioning that the location of TLR9 on the cell membrane plays an essential role in its function and subsequent activation of signaling pathways. For example, TLR9 present on the basolateral membrane of intestinal epithelial cells activates the nuclear factor kappa B (NF-ĸB) pathway compared to the apical membrane that inhibits it [38]. The c-Jun N-terminal kinase (JNK) and NF-κB pathways are essential due to their role in directing the synthesis of pro-inflammatory cytokines [39].

During infection, pro-inflammatory cytokines are produced and released from various cell types, including immune cells, thus initiating cell inflammation. Cytokines can have anti-inflammatory or pro-inflammatory properties. However, some cytokines may promote or suppress inflammation, depending on the immunological situation. Interestingly, anti-inflammatory cytokines are required to inhibit pro-inflammatory cytokine production, consequently suppressing inflammation, whereas pro-inflammatory cytokines are necessary for the activation of immune cell function. Thus, it is evident that a balance between the two has to be maintained in order to avoid immunosuppression or excessive pro-inflammatory response [40]. Many lactic acid bacteria (LAB) produce metabolites that can induce cytokine production against pathogens [41]. For example, inactivated LAB cells have been found to induce the production of various cytokines such as interleukin-12 (IL-12) and IL-10 in macrophages and DCs, respectively [42,43].

Bacterial metabolites such as lactic acids, organic acids, bacteriocins, proteases, peroxides, and exopolysaccharides are known to have antibacterial and antifungal properties [44,45]. Organic acids such as short-chain fatty acids (SCFAs; mainly acetate, propionate, butyrate), formic acid, and propionic acid act against pathogenic bacteria by interfering with the cytoplasmic membrane structure and nutrient transport, as well as by influencing macromolecular synthesis [46]. Nevertheless, the effectiveness of organic acids is primarily determined by the pH. At low pH values, organic acids are found in non-dissociated forms and can penetrate the bacteria’s hydrophobic cell membranes [47]. Once they are inside the bacterial cell, SFCAs dissociate into anions and protons. However, the bacterial cell is trying to export the extra protons since the cytoplasm has a neutral pH, leading to the depletion of cellular energy [48].

Bioactive peptides such as bacteriocins have been reported to display inhibitory activity against various pathogens such as *Listeria monocytogenes*, *Clostridium perfringens*, *Salmonella enterica*, and *Escherichia coli* [49,50]. Bacteriocins can either have a bactericidal or bacteriostatic effect, inhibiting cell growth, and have lately been found to exert an antibiofilm activity [51]. Other antimicrobial molecules released by probiotic bacteria are hydrogen peroxide (H_2_O_2_) and extracellular high molecular weight sugar polymers known as exopolysaccharides (EPS). Hydrogen peroxide produced by lactobacilli may oxidize different compounds (proteins, nucleic acids, and lipids) of pathogenic microorganisms, thus leading to significant impairments in structure and possibly to the loss of cell viability [52,53]. On the other hand, although the mechanism of action is not yet clear, it has been shown that EPS from lactobacilli and bifidobacteria plays an important role in protecting pathogenic bacteria such as enterotoxigenic *E. coli* and *Citrobacter rodentium* [54,55].

### 2.1. Effects against Pathogenic Bacteria

Although it is both preventable and treatable, diarrheal disease is one of the leading causes of death among children under the age of five. One of its major causes is inadequate sanitation or contamination of water that can lead to either bacterial, viral, or parasitic infections [3]. The potential infectious disease targets for postbiotics discussed in the current review are summarized in Table 1.

#### 2.1.1. *Escherichia coli*

Infection with pathogenic strains of *E. coli* is one of the most common etiological agents of moderate-to-severe diarrhea in low-income countries and is considered a significant public health problem [84,85]. Accumulated evidence from in vitro studies supports the use of postbiotics for the protection against infectious diseases. One prominent attempt was made by Abdelhamid and coworkers, showing that six CFS were able to reduce the growth of two multi-resistant clinical *E. coli* isolates (WW1 and IC2) [56]. Furthermore, all six CFS derived from Man–Rogosa–Sharpe (MRS) broth or skim milk were effective in inhibiting the biofilm formation of both *E. coli* strains, except for skim milk CFS that exhibited zero or slight *E. coli* IC2 biofilm inhibition.

A recent study demonstrated that CFS from *Bifidobacterium bifidum* BBA1 and *Bifidobacterium crudilactis* FR/62/B/3 grown in media supplemented with 3′-sialyllactose, a major bovine milk oligosaccharide, induced a significant decrease in the expression of genes mainly implicated in the virulence mechanism of *E. coli* O157:H7 [57]. Another Bifidobacterium-producing postbiotic metabolite that acts against *E. coli* O157:H7 is *Bifidobacterium bifidum* ATCC 29521 [58]. In vitro experiments indicated that CFSM fractions from *Bifidobacterium bifidum* ATCC 29521 were able to reduce the attachment of the pathogen to intestinal cells by 70%. Moreover, heat-killed *Lactobacillus acidophilus* strain LB (*Lactobacillus* Boucard) with its SCS displayed a high adherence to Caco-2 cells and was able to inhibit the cell association and cell entry of different diarrheagenic bacteria including enterotoxigenic (ETEC) and enteropathogenic (EPEC) *E. coli* [59,60,86]. It can be postulated that postbiotics present in the SCS are responsible for the increased adhesion to Caco-2 cells. For instance, the ability of *L. acidophilus* LB to adhere to Caco-2 cells was significantly decreased when the CFS was discarded or replaced by fresh culture medium. This observation suggested that a secreted metabolic product produced by *L. acidophilus* LB was responsible for its increased adhesion to Caco-2 cells. By treating the supernatant with trypsin and pronase, the adhesion of *L. acidophilus* LB was greatly decreased, leading the authors to conclude that this metabolic product was an extracellular protein. In the same study, a second factor, namely, a non-proteinaceous cell surface component, also seemed to mediate the adhesion of *Lactobacillus* strain LB to Caco-2 cells [87]. Recently, the three novel probiotics *Lactobacillus paracasei* (reclassified to *Lacticaseibacillus paracasei*) CNCM I-4034, *Bifidobacterium breve* CNCM I-4035, and *Lactobacillus rhamnosus* (reclassified to *Lacticaseibacillus rhamnosus*) CNCM I-4036 were isolated from the stool of breastfed infants by Muñoz-Quezada and coworkers [83,88]. In addition, the same group evaluated the ability of CFSs derived from the three probiotics to inhibit the growth of *E. coli* ETEC and EPEC [61]. The authors reported that all CFSs were able to inhibit the growth of *E. coli* up to 40%. However, when the CFSs were neutralized, this antimicrobial activity was either maintained or lost. Another study has also reported that the neutralization of CFS from lactobacilli had a negative effect on its antimicrobial activity against *E. coli* ETEC [62].

Another group investigated the potential postbiotic use against neonatal *E. coli* K1 infection [63]. The CFS from *Lactobacillus rhamnosus* GG (LGG) was pre-incubated with Caco-2 cells or administered to neonatal rats and then exposed to *E. coli* [83]. This pre-treatment with the CFS could inhibit adhesion, invasion, and translocation of *E. coli* to Caco-2 monolayer. In the case of neonatal rats, the group exposed to CFS reduced the bacterial intestinal colonization, translocation, dissemination, and systemic infection. Moreover, pre-incubation with CFS could promote the maturation of neonatal intestinal defense as treated rats had higher expression of immunoglobulin A (IgA) compared to untreated rats. Moreover, two recent projects from the same group identified a novel secreted protein (HM0539) from the CFS of LGG that may promote the development of neonatal intestinal defense and prevent against *E. coli* K1 and O157:H7 infection [64,89]. More specifically, HM0539 treatment significantly increased the expression of tight junction proteins and inhibited their destruction by *E. coli* O157:H7 in HT-29 cells and the jejunum of mice. Overall, these findings contribute to the fact that postbiotics instead of probiotics could be good candidates for the prophylactic treatment against *E. coli* K1 and O157:H7 infection, thus potentially protecting against neonatal sepsis and meningitis.

#### 2.1.2. *Cronobacter sakazakii*

Growing concerns over emerging antibiotic resistance coupled with the high risk of *Cronobacter sakazakii* infections in formula-fed infants have led to new studies investigating the efficacy of postbiotics produced by different probiotic strains against *C. sakazakii* [90]. For instance, lyophilized and heat-treated probiotic *Lactobacillus acidophilus* INMIA 9602 Er 317/402 (strain Narine) proved to inhibit the growth of *C. sakazakii* in contaminated reconstructed powdered infant formula after coculture for 6 h at 37 °C [65]. However, when employing the agar well diffusion method, CFS Narine was the most effective in inhibiting *C. sakazakii* growth compared to lyophilized and heat-treated Narine. Indeed, when examining the morphology by transmission electron microscopy, it was evident that CFS Narine was able to damage the cell membrane of *C. sakazakii*. On the other hand, and in agreement with previous studies, when the CFS was neutralized, this in vitro antimicrobial activity was diminished. Four more CFS (filtered and filtered + heat inactivated) from *Lactobacillus casei* strain Shirota (Yakult); *Lactobacillus sporongenes*, *Streptococcus faecalis*, *Clostridium butyricum*, *Bacillus mesentericus* (Bifilac; cells); *Streptococcus faecalis*, *Lactobacillus sporongenes*, *Clostridium butyricum*, *Bacillus mesentericus* (Vibact; spores); and *Lactobacillus sporongenes* (Caplac; spores) were successful in inhibiting *C. sakazakii* growth. The pathogenic potential of this pathogen was also increased due to its high biofilm-forming capacity. Additionally, in this study, a higher biofilm inhibitory activity (> 80%) was observed at the initial stages of biofilm formation. However, the same group reported that when the four CFSs were neutralized, the possessed antimicrobial activity was lost [66].

#### 2.1.3. *Clostridioides difficile*

Postbiotics may also be a new alternative to prevent or treat *Clostridioides difficile* (formerly known as *Clostridium difficile*) infections (CDI). Although considered extremely rare in neonates and infants, further studies report the occurrence of diarrhea in this specific population [91,92]. Routine testing for CDI in infants is currently not recommended due to the high asymptomatic colonization in this group and should be performed only when other causes of diarrhea, such as rotavirus, have been excluded [93,94]. However, toxigenic *C. difficile* has been identified alone or in co-infection with rotavirus, suggesting that it could be a contributing factor to diarrhea in infants [92,95]. New evidence shows that CFS from various lactic acid bacteria (LAB) cocultured with multi-resistant *C. difficile* can reduce the cytotoxic effects of clostridial toxins on different cell lines compared to pure *C. difficile* CFS. Additionally, neutralized CFS from LAB + *C. difficile* can reduce the level of IL-8 and tumor necrosis factor α (TNF-α), two pro-inflammatory cytokines released by intestinal epithelial cells, caused by the CFS of *C. difficile* [67,68].

#### 2.1.4. *Salmonella* spp.

Postbiotics could also be a potential treatment against *Salmonella* infections. CFSs from *Lactobacillus paracasei* CNCM I-4034, *Bifidobacterium breve* CNCM I-4035, and *Lactobacillus rhamnosus* CNCM I-4036 were able to inhibit the growth of *Salmonella typhimurium* and/or *Salmonella typhi* but in most cases this antimicrobial activity was lost when the CFSs were neutralized [61]. In addition, CFS from *Bifidobacterium bifidum* ATCC 29521 proved to inhibit the multiplication of *Salmonella typhimurium* in macrophages, thus affecting its ability to colonize the gastrointestinal tract [58]. Besides interfering with its growth, a study on Caco-2 cells indicated that heat-killed *Lactobacillus acidophilus* LB with its SCS had a protective effect against *Salmonella typhimurium* by means of blocking the cell entry of the pathogen into the cell line [59]. Moreover, a study on a novel organ culture system of human intestinal mucosa showed that the supernatant from *Lactobacillus paracasei* B21060 grown in MRS inhibited the inflammatory potential of *Salmonella* and conditioned the epithelium against *Salmonella* invasion [69].

On the other hand, postbiotics could increase the inflammatory response in the presence of pathogens. One interesting study used an in vitro bilayer system of human intestinal epithelial cells (IEC) and intestinal-like dendritic cells (DC) to study the immunomodulatory properties of *Lactobacillus paracasei* CNCM I-4034 CFS [83]. The authors reported that the CFS increased the production of pro-inflammatory cytokines [IL-1β, IL-6, transforming growth factor β2 (TGF-β2), and interferon-γ-inducible protein 10 (IP-10)] when *Salmonella typhi* was present, thus stimulating the innate immune system. This increase in cytokine production was associated with the downregulation of TLR genes, except TLR9, which was upregulated. Due to increased concerns that probiotics could augment ongoing inflammation, Zagato and coworkers decided to investigate whether postbiotics could be an alternative option in suppressing inflammation. Indeed, the in vitro experiment indicated that when co-incubated with *Salmonella typhimurium*, only the CFS and not the live cells from *Lactobacillus paracasei* CBA L74 could reduce the pro-inflammatory IL-12p70 and increase anti-inflammatory IL-10 in human DCs [43]. Moreover, in the same study, mice treated with *L. paracasei* CBA L74 CFS were protected against dextran sulfate sodium (DSS)-induced colitis since they demonstrated reduced weight loss compared to the mice treated only with live cells. Trying to evaluate the properties of the fermented infant formula by *L. paracasei* CBA L74, the authors found that it displayed protective effects against colitis, showed a slightly longer survival to a lethal dose of *Salmonella*, and increased the production of anti-inflammatory cytokines. On the other hand, the administration of live *L. paracasei* CBA L74 led to the death of the mice. Overall, these results indicated that it is essential to consider the use of postbiotics in infant formulas, especially when targeting their administration to immunocompromised infants. Another animal study was carried out to determine the protective capacity of autochthonous *Lactobacillus* strains to prevent *Salmonella* Typhimurium infection in mice. The strains used in the study were (*Lactobacillus helveticus* (Lh 05, Lh 06, Lh 07, and Lh 08), *Lactobacillus delbrueckii* subsp. *bulgaricus* (Lb 03, Lb 09, Lb 10, Lb 11, and Lb 12), and *L. delbrueckii* subsp. *lactis* (Ll 210 and Ll 133). The cell-free supernatant was spray-dried to inactivate it. The authors reported that the administration of postbiotics was effective in protecting the mice from *Salmonella typhimurium* infection, increasing the survival in the postbiotic group [96].

### 2.2. Effects against Viruses

#### 2.2.1. Influenza

Influenza virus infections contribute significantly to respiratory hospitalizations among children, with those aged less than five years old being at a higher risk for influenza complications [97,98]. This not only has detrimental health outcomes for the children but is considered a financial burden to society, employees, and parents who have to stay home to attend to their children [99,100]. Apart from vaccines and antiviral medications, postbiotics could be a useful alternative for the early treatment of seasonal flu. New evidence reveals that heat-killed *Lactobacillus paracasei* MCC1849 is efficient in increasing the IgA production in the small intestine and serum, facilitating protection against influenza virus infection in mice [81]. IgA plays an essential role in the host defense mechanism as it can inhibit the adherence of pathogenic bacteria and viruses to epithelial cells. Of note, an increased IL-10 gene expression in MCC1849-fed mice compared to the control was confirmed, possibly positively affecting the production of IgA. Finally, heat-killed *Lactobacillus paracasei* MCC1849 increased the gene expression of IL-12p40, IL-10, IL-21, signal transducer and activator of transcription 4 (STAT4), and B cell lymphoma 6 (BCL6) associated with follicular helper T (T_FH_) cell differentiation Peyer’s patches, where IgA production occurs. Additionally, postbiotic compounds produced by *Lactobacillus plantarum* (reclassified to *Lactiplantibacillus plantarum*) YML009 and *Leuconostoc mesenteroides* YML003, two strains isolated from kimchi, exhibited an antiviral effect on various types of influenza virus, including H1N1 and H9N2, respectively [71,72]. More specifically, the antiviral efficacy of *L. plantarum* YML009 CFS was increased when compared with Tamiflu, an antiviral medication, and it was found to revoke viral infection entirely.

#### 2.2.2. Rotavirus

A significant cause of diarrhea-related child mortality worldwide is infection with RV [101]. Apart from *E. coli*, RV is the other most common cause of moderate-to-severe diarrhea, particularly in low-income countries. Some studies have demonstrated that postbiotics are effective in preventing and treating RV diarrhea. For instance, heat-inactivated *L. acidophilus* LB proved to be effective in treating children with diarrhea by decreasing its duration [102,103]. This decrease was more prominent in children that did not receive antibiotics before inclusion. However, other studies found no effect on the duration of diarrhea. A randomized clinical trial compared the impact of viable and heat-inactivated LGG in children under the age of four with rotaviral diarrhea. The findings indicated that the group of children treated with viable LGG had higher sera anti-RV IgA response to rotavirus than children in the postbiotic group. Nevertheless, in the clinical outcome, there were no differences in the duration of diarrhea, which was short (2.5–3 days) and equal between probiotic and postbiotic groups [73]. Another study, conducted in rats, evaluated the effect of a prebiotic-enriched, heat-treated fermented milk formula on the prevention of rotavirus-associated diarrhea [18]. This dietary intervention reduced two of the clinical symptoms of diarrhea (incidence and severity) and improved the immune response against RV by increasing sera anti-RV IgG and intestinal anti-RV IgA. These findings demonstrate that postbiotics, produced during the fermentation of infant formulas, might also have the potential to prevent diarrhea in children.

#### 2.2.3. Human Immunodeficiency Virus

Human immunodeficiency virus (HIV) transmission from mother to child remains a significant public health problem. In particular, in countries of Southern Africa, more than one in five pregnant women are HIV-infected, and additionally, regarding children, infectious diseases are the leading cause of infant mortality [104,105]. Until 2010, the World Health Organization (WHO) advised HIV-positive mothers to avoid breastfeeding and instead use infant formula for their child’s nutrition to prevent transmission of HIV [106,107,108]. Since then, new evidence has surfaced, suggesting that the combination of breastfeeding and antiretroviral treatment can significantly reduce the risk of postnatal HIV transmission to infants through breastfeeding [109]. Over the years, breastmilk use has received growing attention due to its immunomodulatory and antimicrobial properties. More recently, 38 strains of heat-killed commensal breastmilk bacteria and their CFS were evaluated for their capacity to constrain HIV-1 infection in vitro [74]. Findings showed that the isolated strains effectively inhibited HIV-1 infection, with heat-inactivated cells exerting an increased antiviral activity compared to the CFS. This might be due to the association of the virus with bacterial surface components, such as peptidoglycans, exopolysaccharides, and lipopolysaccharide moieties. Consequently, postbiotics from human breastmilk may be essential for protecting against HIV-1 in the breastfeeding infant.

### 2.3. Effects against Candida *spp.*

Another major cause of pediatric-associated infections is the prevalence of candidiasis. In the United States, invasive candidiasis in the pediatric population mainly occurs in neonates and infants less than one year of age and can often lead to high mortality rates [110]. Prematurity and admission to the intensive care unit are considered the main predisposing factors for candidiasis [111]. Prevention against fungal infection in preterm infants can be achieved through antifungal medication, but there are strong concerns over potential adverse effects and the possible emergence of resistant strains [112]. In light of this, researchers have focused on finding alternatives, and through in vitro studies have demonstrated that SCS of *L. rhamnosus* and *L. casei* exhibit antifungal activity against blastoconidia and biofilm of *C. albicans* [77]. Moreover, CFS from honey and vaginal LAB were proven to be interesting sources of antifungals [75,76]. Compounds present in the supernatant inhibited the adhesion and decreased the biofilm formation of five *Candida* spp. on polystyrene and HeLa cells. It can be hypothesized that these compounds could help prevent adhesion on medical devices, thus decreasing the risk of potential candidiasis in hospitalized children.

### 2.4. Effect against Common Pediatric Infectious Diseases with Unknown Cause

Postbiotics from *L. paracasei* CBA L74 derived from fermented formula can also prevent common pediatric infectious diseases. For instance, Corsello and coworkers reported that when the fermented product was given to healthy children aged 1–4 years in daycare or preschool, its consumption was associated with a reduction in the incidence of common infectious diseases [17]. However, the cause of pediatric infectious diseases was not evaluated. Additionally, the same product induced an increase in fecal biomarkers of innate (α- and β-defensins, cathelicidin) and acquired immunity (secretory IgA) after 3 months. Cow’s milk or rice fermented with *L. paracasei* CBA L74 was also found to prevent common pediatric infectious diseases in children attending daycare after 3 months of consumption [78]. This could be explained by the higher levels of α-defensin, β-defensin, cathelicidin, and IgA in stools that were higher in cow’s milk and rice group compared to the placebo group. These findings underline the importance of fermented foods and their role in the modulation of innate and acquired immunity.

Another recently developed infant formula fermented with *Bifidobacterium breve* C50 and *Streptococcus thermophilus* 065 was assessed for its preventive effect against the incidence of acute diarrhea in healthy infants aged 4–6 months [79]. While consumption lasted for 5 months, the outcome showed that there was no difference on the incidence and duration of diarrhea episodes between the control (standard infant formula) and the fermented formula. However, the fermented formula was shown to alleviate the severity of diarrhea episodes.

### 2.5. Effects against Neonatal Necrotizing Enterocolitis

Prematurity is considered a predisposing factor for necrotizing enterocolitis (NEC), a disease with a 30% mortality rate in extremely and very low birth weight infants [113]. Although the pathogenesis of this disease is not fully understood, scientific evidence points out that dysbiosis is playing an important role [114]. Thus far, a limited amount of clinical trials using postbiotics have been conducted, but findings from in vitro or in vivo studies look promising. For instance, a group of scientists reported that DNA derived and released from inactivated *Lactobacillus rhamnosus* HN001 was sufficient in attenuating NEC severity in newborn mice and premature piglets [82]. Interestingly, it was shown that activation of TLR9 by *L. rhamnosus* HN001 DNA was an important mediator for the protection against NEC. Over the years, clinical studies have shown that the administration of bifidobacteria could prevent NEC development in premature infants [115]. It is also important to mention that various studies have linked clostridia with the development of NEC [116,117]. Therefore, postbiotics derived from whey cow’s milk retentate fermented by bifidobacteria might be effective in decreasing intestinal clostridia [94]. The difference in the gut microbiome of preterm infants compared to the one of term infants could be considered an important factor in NEC development [118]. Since concerns over the development of sepsis in infants after probiotic administration are raised, the use of postbiotics instead of probiotics in this vulnerable group might be a useful strategy in shaping their gut microbiome and thus preventing or treating NEC [119,120,121]. However, it is essential to add that specific probiotics with documented efficacy are recommended for preventing NEC by both the European Society for Paediatric Gastroenterology Hepatology and Nutrition (ESPGHAN) and the American Gastroenterological Association (AGA) [122,123]. Over the years, a different array of postbiotic compounds, including reuterin; SCFAs; tryptophan; and various organic acids, such as taurine, histamine, and spermine, are found to either inhibit bacterial growth or promote activation of immune responses [124,125]. In contrast, another study focused on the use of postbiotics and their impact on the gut microbiota composition of preterm infants. According to Campeotto and coworkers, heat-treated fermented preterm infant formula by *Bifidobacterium breve* C50 and *Streptococcus thermophilus* 065 did not significantly modulate the bacterial colonization, but it was well tolerated, meaning that infants did not lose weight [126]. This is an important finding as reduction in weight gain can negatively affect the neurodevelopment of preterm infants [127]. Finally, in the same study, the fermented formula reduced the fecal calprotectin (increased levels of calprotectin are associated with inflammatory gastrointestinal diseases in children) and increased secretory IgA, suggesting immunomodulatory properties [126,128,129].

## 3. Discussion

Currently, the majority of the studies describing mechanisms of postbiotic action have been performed in vitro or in animal models by focusing only on mechanisms targeting either the pathogens or the host. Therefore, considerable research is required to establish the clinical relevance of postbiotics. In addition, clinical studies targeting the mechanism behind the postbiotic effect on host–pathogen interaction are required to understand the effects of postbiotics in humans.

Overall, the majority of clinical studies that tested postbiotic administration for the prevention of common infectious diseases in children did not find any significant differences in adverse effects between the intervention and placebo groups [17,78,79]. Additionally, when postbiotics were used for the treatment of mild RV diarrhea in children, no major side effects were noted [103]. However, there is a need for more randomized controlled trials that focus on a specific metabolite or component and establish its effectiveness and safety for preventing common pediatric infectious diseases measuring a pre-specified clinical outcome. Besides safety and effectiveness concerns, another challenge is the lack of a consensus regarding the definition of postbiotics that delays their commercialization and regulation [26]. Agreeing on a proper definition will also help increase their study and allow health benefits to be linked to different classes of postbiotics.

Besides pathogen infections, childhood obesity is another disease with increasing prevalence, especially in developed countries, but that is very challenging in its management. Promising preliminary studies show that SCFAs may play a role in energy metabolism and stimulate secretions of hormones that enhance food absorption [130]. However, the role of SCFAs in obesity still remains controversial due to mixed results from clinical studies [131,132]. On the other hand, in developing countries, malnutrition affects many children that become predisposed to infections [133]. A promising strategy targeting alterations in the functional activity of the immune system in malnourished children could be bioactive compounds that regulate host immune responses. Recently, researchers from Mexico showed that pre-treatment with heat-killed *L. casei* IMAU60214 can induce cytokine secretion on monocyte-derived macrophages isolated from malnourished children [134]. This is a positive first step on elucidating the role of postbiotics in malnutrition management, but more studies are needed. Due to their immunomodulatory properties, postbiotics could also be explored as a novel therapeutic approach for the prevention and treatment of severe acute respiratory syndrome coronavirus 2 (SARS-CoV-2). A recent study proposed the role of the microbiome in the development of severe cases of coronavirus disease 2019 (COVID-19) [135], while others suggested that metabolites produced by the intestinal microbiome could be used for developing a drug for individuals who are susceptible to SARS-CoV-2 infection [136,137]. However, well controlled studies in humans are urgently needed to demonstrate whether postbiotics would have any effect against these diseases.

## 4. Conclusions

In summary, recently accumulated evidence from cell cultures and animal models indicates that postbiotics may be a promising strategy to prevent or protect against infectious diseases in children under the age of five. Postbiotics can exert immunomodulatory, antimicrobial, and antibiofilm effects similar to their parent probiotics; have a longer shelf life; and could be introduced in developing countries where certain infectious diseases are more prevalent. However, further studies are necessary to characterize different postbiotic compounds and to shed more light on their exact mechanisms of action and the way in which they protect the host from diseases. Finally, even if current findings from in vitro and in vivo experiments support the use of postbiotics, more clinical trials are required to validate their protective effects against infectious diseases.

## Figures and Tables

**Table 1:** Potential infectious disease targets for postbiotics based on current evidence.

Targeted Pathogen	Probiotic Bacteria	Preparation	Type of Study	Bioactivity or Effect	Reference
*Escherichia coli*	*Lactobacillus acidophilus* EMCC 1324 (La) *, *Lactobacillus helveticus* EMCC 1654 (Lh), *Lactobacillus plantarum* ss. *plantarum* EMCC 1027 (Lp), *Lactobacillus rhamnosus* EMCC 1105 (Lr), *Bifidobacterium longum* EMCC 1547 (BL), *Bifidobacterium bifidum* EMCC 1334 (Bb)	Cell-free supernatant (CFS; filtered) of MRS or milk fermented by probiotics	In vitro	CFS of MRS fermented by all probiotics resulted in inhibition of two multiresistant *E. coli* (WW1 and IC2) biofilm formation; CFSM of milk fermented by *B. longum* and *L. helveticus* reduced *E. coli* WW1 biofilm	[56]
	*Bifidobacterium bifidum* BBA1 and *Bifidobacterium crudilactis* FR/62/B/3	Cell-free supernatant (CFS; filtered) from probiotics grown in media supplemented with 3′-sialyllactose (major bovine milk oligosaccharide)	In vitro	Both CFSs induced a significant decrease in virulence gene expression of *E. coli* O157:H7	[57]
	*Bifidobacterium bifidum* ATCC 29521	Cell-free supernatant (CFS; filtered) of modified MRS fermented by probiotic	HeLa and macrophage cell lines	Downregulated virulence genes in *E. coli* O157:H7; inhibited its adhesion to HeLa cell line	[58]
	*Lactobacillus acidophilus* LB	Heat-inactivated probiotic with its spent culture supernatant (SCS)	Caco-2 cell culture	Inhibited *E. coli* ETEC adhesion	[59]
	*Lactobacillus acidophilus* LB	Heat-inactivated probiotic with its spent culture supernatant (SCS)	Caco-2 cell culture	Inhibited cell association and cell invasion of *E. coli* ETEC, DAEC, and EPEC	[60]
	*Lactobacillus paracasei* CNCM I-4034, *Bifidobacterium breve* CNCM I-4035, *Lactobacillus rhamnosus* CNCM I-4036)	Cell-free supernatant (CFS; filtered) of MRS fermented by probiotics	Monitoring bacterial growth	Inhibited the growth of *E. coli* ETEC and EPEC	[61]
	*Lactobacillus acidophilus* RY2, *Lactobacillus salivarius* MM1, *Lactobacillus paracasei* En4	Spent culture supernatant (SCS) from bacteria grown in MRS + cystein	Colony count assay	Inhibited growth of *E. coli* ETEC	[62]
	*Lactobacillus rhamnosus* GG	Cell-free supernatant (CFS; filtered) of MRS fermented by probiotic	Caco-2 cell culture	Blocked adhesion, invasion, and translocation of *E. coli* K1	[63]
	*Lactobacillus rhamnosus* GG	Novel secreted protein (HM0539) present in the CFS	Infected Sprague-Dawley neonatal rats	Promoted the development of neonatal intestinal defense and prevented against *E. coli* K1 pathogenesis	[64]
*Cronobacter sakazakii*	*Lactobacillus acidophilus* INMIA 9602 Er 317/402 strain Narine	Heat-inactivated probiotic	Agar well diffusion method	Inhibited the growth of *C. sakazakii* in contaminated reconstructed powdered infant formula	[65]
	*Lactobacillus casei* strain Shirota (Yakult); *Lactobacillus sporongenes*, *Streptococcus faecalis, Clostridium butyricum, Bacillus mesentericus* (Bifilac; cells); *Streptococcus faecalis*, *Lactobacillus sporongenes, Clostridium butyricum, Bacillus mesentericus* (Vibact; spores); *Lactobacillus sporongenes* (Caplac; spores)	Cell-free supernatant (CFS; filtered) from isolated probiotics	In vitro	Four probiotic-derived CFS (filtered and filtered + heat inactivated) possessed antimicrobial activity against *C. sakazakii*; a higher biofilm inhibitory activity (> 80%) was observed at initial stages of biofilm formation	[66]
*Clostridioides difficile*	*Enterococcus faecium, Lactococcus lactis*	Cell-free supernatant (CFS; filtered) of MRS fermented by lactic acid bacteria	Caco-2 cell culture	Diminished the expression level of proinflammatory cytokines induced by the cell-free supernatant of *C. difficile*	[67]
	*Lactobacillus rhamnosus* GG	Cell-free supernatant (CFS; filtered) and cell lysate (sonicated and filtered) of co-cultured toxigenic *C. difficile* with LGG	Vero cell culture	Decreased the cytotoxic effect of both extracellular and intracellular clostridial toxins resulting in up to 30% increase in cell viability	[68]
*Salmonella* spp.	*Bifidobacterium bifidum* ATCC 29521	Cell-free supernatant (CFS; filtered) of modified MRS fermented by probiotic	HeLa and macrophage cell lines	Downregulated virulence genes in *S.* Typhimurium; inhibited its adhesion to HeLa cell line; diminished the ability of *Salmonella* to survive and multiply within macrophages	[58]
	*Lactobacillus acidophilus* LB	Heat-inactivated probiotic with its spent culture supernatant (SCS)	Caco-2 cell culture	Inhibited cell association and cell invasion of *S. typhimurium*	[60]
	*Lactobacillus paracasei* CNCM I-4034, *Bifidobacterium breve* CNCM I-4035, *Lactobacillus rhamnosus* CNCM I-4036)	Cell-free supernatant (CFS; filtered) of MRS fermented by probiotics	Monitoring bacterial growth	Inhibited the growth of *Salmonella typhimurium* and/or *Salmonella typhi*	[61]
	*Lactobacillus paracasei* B21060	Supernatant of lactic acid bacteria grown in MRS	Ex vivo organ culture model	Inhibited the inflammatory potential of *Salmonella* and conditioned the epithelium against *Salmonella* invasion	[69]
	*Lactobacillus paracasei* CNCM I-4034	Cell-free supernatant (CFS; filtered) of MRS fermented by probiotics	Coculture of dendritic and Caco-2 cells	Increased the production of pro-inflammatory cytokines in the presence of *Salmonella typhi*	[70]
Influenza	*Lactobacillus plantarum* YML009	Cell-free supernatant (CFS; filtered) of MRS fermented by lactic acid bacteria	MDCK cells and hemagglutination assay	Displayed a significant antiviral activity against influenza virus H1N1 and was more effective than Tamiflu	[71]
	Leuconostoc mesenteroides YML003	Cell-free supernatant (CFS; filtered) of MRS fermented by lactic acid bacteria	MDCK cells and hemagglutination assay	Demonstrated antiviral activity against low-pathogenic avian influenza (H9N2)	[72]
Rotavirus	*Bifidobacterium breve* C50 and *Streptococcus thermophilus* 065	Heat-treated fermented milk containing the prebiotic mixture scGOS/lcFOS	Rotavirus-infected Lewis rats	Reduced the incidence and severity of the rotaviral diarrhea; enhanced the host’s immune system	[18]
	*Lactobacilus rhamnosus GG*	Orally administered heat-inactivated probiotic	Children under the age of 4 with rotavirus diarrhea	Rotavirus diarrhea recovery was equal for both viable and heat-inactivated probiotic-receiving groups	[73]
Human immunodeficiency virus	Lactic acid bacteria isolated from human milk	Heat-inactivated lactic acid bacteria	TZM-bl cells	Significantly inhibited R5-tropic HIV-1 infection	[74]
*Candida* spp.	Four LAB isolated from honey (*Lactobacillus plantarum* HS, *Pediococcus acidilactici* HC, *Lactobacillus curvatus* HH, and *Pediococcus pentosaceus* HM)	Cell-free supernatant (CFS; filtered) of MRS fermented by probiotics	Microtiter plate method	Decreased the biofilm formation by *Candida* spp.	[75]
	*Lactobacillus crispatus* B1-BC8, *Lactobacillus gasseri* BC9-BC14, *Lactobacillus vaginalis* BC15-BC17	Cell-free supernatant (CFS; filtered) of MRS fermented by probiotics	HeLa cells	Reduced the adhesion of *Candida* spp.	[76]
	*Lactobacillus casei* ATCC 334, *Lactobacilus rhamnosus* GG (ATCC 53103)	Cell-free supernatant (CFS; filtered) of MRS fermented by lactic acid bacteria	Kirby–Bauer disk diffusion susceptibility test	Exhibited antifungal activity against blastoconidia and biofilm of *Candida albicans*	[77]
Common pediatric infectious diseases with unknown cause	*L. paracasei CBA L74*	Heat-treated fermented cow’s skim milk powder	Healthy children aged 1–4 years in daycare or preschool	Consumption was associated with a reduction of common infectious disease	[17]
	*L. paracasei CBA L74*	Heat-treated fermented cow’s milk or rice	Healthy children aged 1–4 years attending daycare or preschool	Prevented common pediatric infectious diseases in children after 3 months of consumption	[78]
	*Bifidobacterium breve* C50, *Streptococcus thermophilus* 065	Heat-treated fermented infant formula	Healthy children aged 4–6 months	Did not prevent the incidence of acute diarrhea in healthy infants but reduced its severity	[79]
Necrotizing enterocolitis	Six human strains of *Bifidobacterium breve*	Whey retentate from fermented cow’s milk	SPF C3H male mice, healthy volunteers aged 21–35 years	Led to a decrease in clostridia, bacilli, *B. fragilis*, and fecal pH, as well as to an increase in bifidobacteria	[80]
	*Lactobacillus paracasei* CBA L74	Heat-treated fermented milk powder	C57/BL6 mice	Strong anti-inflammatory activity *in vitro* and protected against colitis or enteric pathogens in vivo	[43]
	*Lactobacillus paracasei* MCC1849	Orally administered heat-inactivated probiotic	Male SPF BALB/c mice	Enhanced antigen-specific IgA secretion and induced follicular helper T cells	[81]
	*Lactobacillus rhamnosus* HN001	Orally administered UV-killed probiotic	Newborn mice and premature piglets	Attenuated necrotizing enterocolitis severity	[82]

* Of note, the genus of *Lactobacillus* has been recently reclassified into 25 genera, which includes 23 novel genera [83].
